# Copolymerization of Propylene with Higher α-Olefins by a Pyridylamidohafnium Catalyst: An Effective Approach to Polypropylene-Based Elastomer

**DOI:** 10.3390/polym12010089

**Published:** 2020-01-03

**Authors:** Fei Yang, Xiaoyan Wang, Zhe Ma, Bin Wang, Li Pan, Yuesheng Li

**Affiliations:** 1 Tianjin Key Laboratory of Composite & Functional Materials, School of Materials Science and Engineering, Tianjin University, Tianjin 300350, China; fyang@tju.edu.cn (F.Y.); zhe.ma@tju.edu.cn (Z.M.); lilypan@tju.edu.cn (L.P.); ysli@tju.edu.cn (Y.L.); 2 State Key Laboratory of Organometallic Chemistry, Shanghai Institute of Organic Chemistry, Chinese Academy of Sciences, 345 Lingling Lu, Shanghai 200032, China

**Keywords:** polyolefin elastomer, polypropylene, higher α-olefin, elastic recovery

## Abstract

In this contribution, we explored the copolymerization of propylene with higher α-olefins, including 1-octene (C8) 1-dodecene (C12), 1-hexadecene (C16) and 1-eicosene (C20), by using a dimethyl pyridylamidohafnium catalyst. A series of copolymers with varied comonomer incorporation, high molecular weight and narrow molecular weight distribution were obtained at mild conditions. The effects of the insertion of the comonomers on the microstructure, thermal and final mechanical properties were systemically studied by ^13^C NMR, wide-angle X-ray scattering, DSC and tensile test. Excellent mechanical performances were achieved by tuning the incorporation and chain length of the higher α-olefins. When the comonomer content reached above 12 mol.%, polypropylene-based elastomers were obtained with high ductility. A combination of excellent elastic recovery and flexibility was achieved for the P/C16 copolymers with about 20 mol.% monomer incorporation. The monomer incorporation and side chain length played a crucial role in determining the mechanical property of the outstanding polypropylene-based elastomers.

## 1. Introduction

Polyolefin represented by polyethylene (PE) and polypropylene (PP) is one of the most important kinds of thermoplastics in the market. It enjoys tremendous production volume and wide applications in our daily life because of their unique combination of chemical and physical properties [1,2,3,4,5]. In recent years, the further prosperity of polyolefin material is motivated by thermoplastic elastomer (TPE) in high value-added applications. Because of their excellent recyclability and processability, TPE is widely used as a very attractive alternative to vulcanized rubbers in many applications [6,7]. Among various developed TPEs, polyolefin elastomer (POE) exhibits many excellent performances, such as low density, good tolerance with aging and ultraviolet, easy processability and low costs. Thus, copolymerization of ethene or propylene with α-olefins, as the most direct and effective approach to POE, has been intensively studied in the past decades [8,9] The properties of the POE, such as strength, stiffness, elasticity, processability and solvent resistance, can be modulated in a wide range by controlling the microstructure of polymer chain, including monomer structure, incorporation and distribution, as well as the molecular weight. The widely tunable properties enable the POE materials to be extensively applied in various fields ranging from elastomer products, adhesive, toughening modifier to compatibilizer.

Since the first commercial POE developed by the copolymerization of ethene and 1-octene, many kinds of olefin-based elastomers with different multi-block or graft structures have been prepared by using different catalytic systems or synthetic strategies [10,11,12,13,14]. In these olefin-based elastomers, the crystalline PE chain domains (hard segments) acting as physical cross-links is indispensable for good strength, while the amorphous ethene/α-olefin chain domains (soft segments) endows the POE with an excellent elastic recovery [15,16]. In this context, the PP-based elastomer is more preferential because PP has a higher melting temperature (*T*_m_) and a lower chain conformational mobility compared to PE, which can reduce the viscous flow and chain entanglement during stretching to some extent [17,18,19]. In addition, PP has two highly ordered microstructures: Isotactic PP (*i*PP) and syndiotactic PP (*s*PP). Integrating these ordered microstructures in the atactic PP (*a*PP) or other amorphous copolymer will develop novel POEs with structural diversity and tunable properties, for example, *s*PP-*b*-poly(ethylene-*co*-propylene)-*s*PP triblock copolymer, *i*PP-*b*-*a*PP-*block(b)*-*i*PP multi-block copolymer, poly(ethylene-*co*-propylene)-*graft(g)*-*i*PP and poly(ethylene-*co*-propylene)-*g*-*i*PP copolymer. These new PP-based elastomers showed outstanding performance, including remarkable elastic properties, excellent stiffness-flexibility balance and easy processability [20,21,22]. With the development of group IV single-site catalysts, we can easily control the chain microstructure and the final material properties [23]. For instance, a series of closely related *ansa*-bridged metallocene-based catalysts afford several grades of *i*PP materials with different levels of stereo-error incorporation in a single reactor process [24,25,26,27,28,29]. Correspondingly, stereo multi-block copolymers could be prepared in one-pot by an “oscillating” catalyst or living catalyst system. These *i*PP-based materials exhibited appealing physical properties ranging from stiff plastics to thermoplastic elastomers. However, it is a key challenge for the development of high-performance PP-based elastomer given that per living catalyst molecule only producing one polymer chain, resulting in low economic efficiency. Moreover, tailoring these polymer microstructures usually requires a very profound insight into the relationships between catalytic performance and chain microstructure, final mechanical properties.

Copolymerization of propylene with other comonomers is a versatile and effective method to modify polypropylene chain and the final properties, which produces a wide range of interesting materials [8,13,30,31,32,33,34]. The performance of the PP-based elastomers is determined by comonomer structure, incorporation and distribution along the backbone. Many works clearly demonstrated that the length and incorporation of side chain remarkably affected the chain entanglement, crystallization of PP backbone, the glass transition temperature (*T*_g_) of propylene copolymers, and the final mechanical properties [35,36,37,38]. So far, 1-hexene (C6) and 1-octene (C8) are two types of the most widely used comonomers for PP-based elastomer, which typically process poor elastic recovery [39,40,41,42,43,44,45,46,47,48]. In contrast, copolymerization of propylene with higher α-olefins not only can provide microstructure diversity, but also can offer excellent physical performance like good stiffness-flexibility balance. However, to the best our knowledge, there are few reports on the copolymerization propylene with the higher α-olefins, such as C12), C16 and C20 [49,50]. To achieve novel PP-based elastomers with excellent performance for high value-added prospective applications, in this work, we focus on the development of elastomeric materials from the copolymerization propylene with higher α-olefins, such as C12, C16 and C20.

**Scheme 1:**
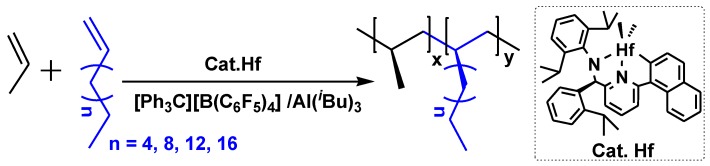
Synthesis of polypropylene-based elastomers with the dimethyl (pyridylamido)hafnium catalyst.

Copolymerization of propylene with higher α-olefins by metallocene catalysts has received extensive attention in recent years [51,52,53]. However, increasing loading dosage of the higher α-olefins generally results in a decrease in the polymerization activity and the molecular weight of the resultant copolymer, preventing further investigation into mechanical properties. Recently, we reported the homo-polymerization of higher α-olefin by the (pyridyl-amido) hafnium catalyst and elaborated the structure-property relationships of the resultant polyolefins [54]. This preliminary work motivated us to further investigate the structure-property relationships of propylene/higher α-olefin copolymers obtained by the versatile and robust (pyridyl-amido)hafnium catalyst as illustrated in Scheme 1. In present work, the copolymerization of propylene with 1-dodecene (C12), 1-hexadecene (C16) and 1-eicosene (C20) are conducted under optimized conditions to prepare the PP-based elastomers. The copolymerization behaviors, the characterization of microstructure and final mechanical properties will be described. The relationship between incorporation and chain length and final physical performance will be discussed systemically as well.

## 2. Experimental Section

### 2.1. General Procedure and Materials

All work involving air- and/or moisture-sensitive compounds was carried out under a nitrogen atmosphere by using standard Schlenk technique. The dimethyl (pyridylamido)hafnium precatalyst was prepared following the procedure reported previously [33,34]. [Ph_3_C] [B(C_6_F_5_)_4_] and Al*^i^*Bu_3_were purchased from Albemarle Corporation and Aldrich, respectively, and used without further purification. Anhydrous solvents used in this work were purified by a Solvent Purification System purchased from Mbraun (Munich, Germany). All the higher α-olefins were dried over CaH_2_ before distillation. It should be noted that for 1-eicosene, a vacuum distillation equipped with air condenser was applied and the fraction was collected at the temperature around 190–210 °C under vacuum.

### 2.2. General Procedure for Copolymerization

The copolymerization was carried out in a 50 mL Schlenk flask under propene atmosphere. The flask was repeatedly evacuated and refilled with propene. The prescribed amount of Al*^i^*Bu_3_, α-olefin and solvent were successively added to the Schlenk flask stirred for 5 min, and the polymerization reaction was started by adding the prescribed amount of catalyst and [Ph_3_C] [B(C_6_F_5_)_4_]. The reaction mixture was stirred at 25 °C. After the desired time, the reactor was vented. The resulted copolymers were precipitated from acidic ethanol, filtered, washed three times with ethanol and acetone, and then dried in vacuo at 40 °C to a constant weight.

### 2.3. Preparation of ESI-MS Sample

Following the procedure described above, the copolymerization of propylene with 1-octene was conducted under the reaction conditions: Catalyst 10 μmol, [Ph_3_C] [B(C_6_F_5_)_4_] 20 μmol, Al*^i^*Bu_3_ 0.20 mmol, propylene 1 atm, *V*_total_ = 40 mL, copolymerization for 0.5 min, 1-octene 6.0 mmol. After the precipitated copolymer was isolated by filtration, the filtrate was concentrated before analyzed by ESI-MS.

## 3. Characterization

The ^13^C NMR data of the polymers were obtained on a Bruker 400 MHz spectrometer at 125 °C, with *o*-C_6_D_4_Cl_2_ as a solvent. Absolute molecular weight, molecular weight distribution, and intrinsic viscosity were obtained by size exclusion chromatography (SEC) with triple detectors. The chromatographic system consisted of a Polymer Laboratories PL 220 high-temperature chromatograph equipped with a two-angle laser light scattering detector (TALLS), a viscosity detector, and a differential refractive index detector. Polymer solutions were prepared with amounts of about 20 mg of polymer in 10 mL of 1,2,4-trichlorobenzene containing a small amount of antioxidant 2,6-di-tert-butyl-4-methylphenol (BHT), and were eluted at 150 °C with a flow rate of 1 mL/min. Electrospray mass spectrometry (ESI-MS) analysis was performed using a Finnigan LCQ ion trap mass spectrometer (Finnigan, San Jose, CA, USA). The samples were dissolved using acetonitrile.

Polymer melting and crystallization temperatures (*T*_m_ and *T*_c_) were measured by differential scanning calorimetry (DSC) using a TA Instruments Q2000 calorimeter (Castle, DE, USA). equipped with an automated sampler. Analyses were performed in crimped aluminum pans under nitrogen and data were collected with the heat/cool/heat cycle at a heating rate of 10 °C min^−1^, and processed with TA Q series software (Castle, DE., USA). Then, the *T*_m_ was obtained from the second heating cycle.

Tensile tests were performed on a screw-driven universal testing machine (Instron 1211, Canton, MA, USA) equipped with a 10 kN electronic load cell and mechanical grips. The tests were conducted at room temperature using a crosshead rate of 20 mm/min. All tests were carried out according to the ASTM standard, and the data reported were the mean and standard deviation from five determinations. The elastic recovery was determined on a portable tensile test machine (TST350, Linkam, UK). The mechanical processing was performed as follows: Tensile cyclic processing was conducted stepwise at room temperature to progressively higher tensile strains. In each step, once the sample reached the appropriate tensile strain, the crosshead direction was reversed, and the sample strain was decreased at the same strain rate. Once the stress was released, the crosshead was immediately reversed, and the sample was then extended again until it reached the next targeted maximum strain. The cyclic mechanical processing was continued until, in the final cycle, the targeted final strain was reached.

## 4. Results and Discussions

### 4.1. The Copolymerization of Propylene with Higher α-Olefins by Dimethyl (pyridyl-amido)Hafnium Catalyst

The copolymerization reactions of propylene with higher α-olefins, including 1-octene, 1-dodecene, 1-hexadecene and 1-eicosene, were conducted under the same conditions by the (pyridyl-amino) hafnium catalyst in the presence of [Ph_3_C] [B(C_6_F_5_)_4_] and Al*^i^*Bu_3_, which is well-known for the excellent ability to incorporate higher α-olefins [37]. The polymerization results are summarized in Table 1. The catalytic activity is retained even at a higher α-olefin feeding concentration up to ca. 6 × 10^7^ g_polymer_⋅mol_Hf_^−1^⋅h^−1^. The molecular weights (*M*_W_s) of the resultant copolymers (300–1700 kg/mol) increased gradually with the increase of comonomer feeding dosage. This is very different from the case of propylene/higher α-olefins copolymerizations catalyzed by the metallocene catalyst, in which a raise of content or chain length of higher α-olefins usually decreases the catalytic activity and the *M*_W_s of the resultant copolymers [55,56]. The monomer incorporation in the polymer chain increased almost linearly with the feeding content, irrelevant to the chain length of the higher α-olefins. In the following analysis, P/C8, P/C12, P/C16 and P/C20 were used to represent the PP-based elastomers containing C8, C12, C16 and C20 comonomer units.

It is noted that the Hf catalyst promoted the propylene/α-olefins copolymerization with multiple catalytical active species, but they produce copolymers with unimodal and relatively narrow polydispersity. It was reported that the ethene/α-olefin copolymers from the (pyridyl-amido) hafnium catalysts had broad molecular weight distributions that can be fit to a bimodal distribution, because either ethene or α-olefin could be inserted into the Hf-naphthyl bond and generated two types of highly active species [57,58,59]. However, under the optimized reaction conditions, all the resultant propylene/α-olefin copolymers possess unimodal molecular weight distributions (*M*_w_/*M*_n_ < 1.6). The ESI-MS analysis was employed to figure out the type and content of the active species in the propylene/α-olefin copolymerization. All of the metal complexes decomposed to the modified ligands, when the MS test was conducted in a humid and aerobic atmosphere. The spectrum clearly showed that the original, propylene-modified and 1-octene-modified ligands (*m*/*z* is 513, 555, and 625, respectively) were present with a ratio of 37:60:3 in the copolymerization of propylene and 6.00 mmol 1-octene (Figure S1 (Supplementary Materials) in supporting information). This result showed that the propene-modified cation complex was the main active species, and the higher α-olefin-modified active species is only about 4.8% of all modified species. The catalytic activity of propene-modified and 1-octene modified active species is still difficult to be directly determined [57,58,59]. In this work, the unimodal distribution of copolymers did not change with the increase of α-olefin mole fraction in the reactor. However, we observed a slightly broadened molecular weight distribution (from 1.6 to 2.2), when prolonging the reaction time (15 min) and loading more 1-octene (10 mmol). This result may be ascribed to the activity differences between the propene-modified and 1-octene modified active species.

### 4.2. Microstructure and Thermal Behavior

**Figure 1:**
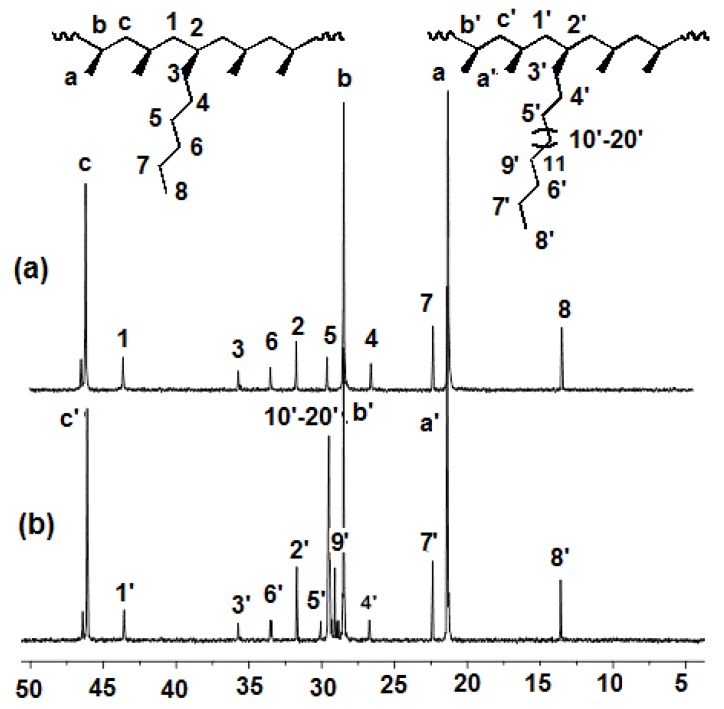
^13^C NMR spectra for: (**a**) P/C8 (1-octene 12.2 mol.%, Table 1, entry 4) and (**b**) P/C20 (1-eicosene 12.2 mol.%, Table 1, entry 16).

**Figure 2:**
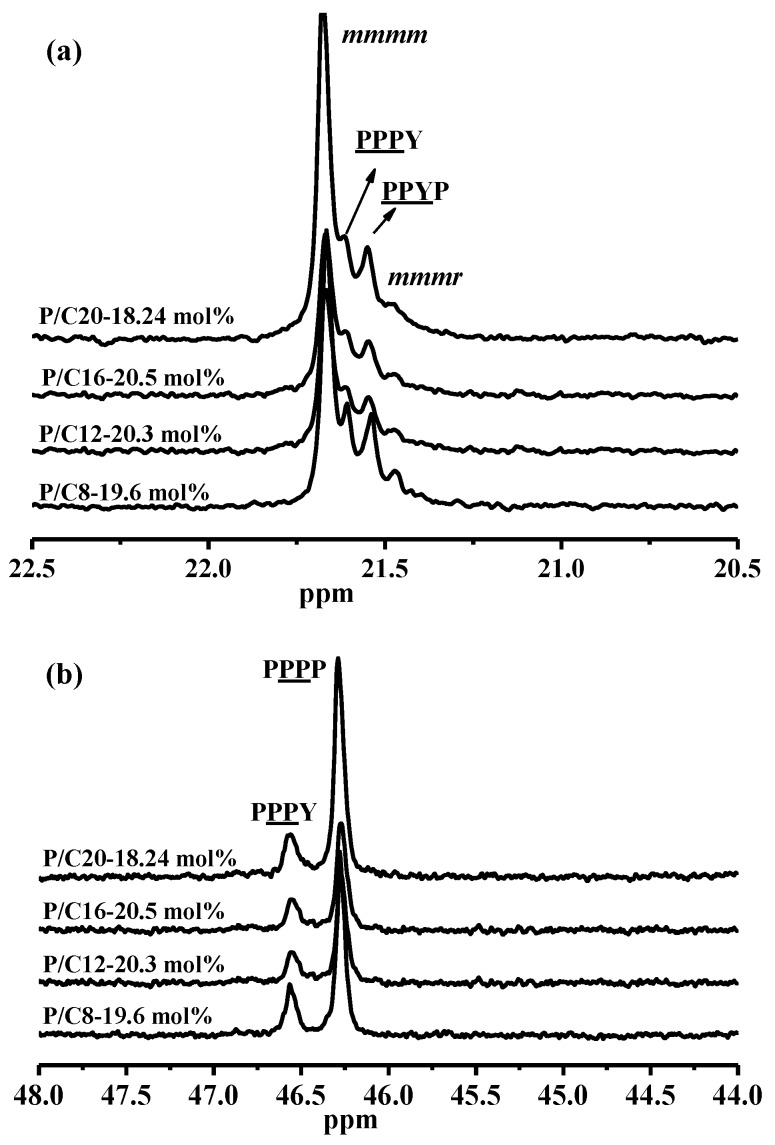
^13^C NMR spectra of the methyl region (**a**) and the methylene region (**b**) of propylene-based elastomers produced by dimethyl (pyridyl-amino) hafnium catalyst.

The microstructures of the typical poly (propylene-*co*-α-olefin) copolymers with different comonomer units were analyzed by the ^13^C NMR spectra. As examples, the ^13^C NMR spectra of P/C8 and P/C20 with about 12 mol.% monomer incorporation are shown in Figure 1. The three main peaks P_1_, P_2_ and P_3_ (at 46.0, 28.4 and 21.4 ppm, respectively) are assigned to isotactic propylene units, and the rest peaks corresponding to side chains with different lengths can also be clearly assigned. The ^13^C NMR analysis suggests that the isotacticity of the copolymer is high up to 95% obtained by the (pyridyl-amino) hafnium when the monomer incorporation is less than 20%. Interestingly, we observed some unknown signal assignments around at methyl region of the polypropylene in copolymers with about 20% monomer incorporation. To describe the monomer sequence distributions, the methyl and methylene regions in the spectra of the P/C20 copolymers with about 20 mol.% comonomer content were analyzed (Figure 2). Here, “P” and “Y” are used to indicate the propylene and α-olefin monomer units, respectively. By inspection of the methyl region in the spectra, the pentads of *mmmm* and *mmmr* at 21.67 and 21.47 ppm was readily assigned, respectively (Figure 2a). Besides, the resonance peaks corresponding to PPPY and PPYP sequences were also detected at 21.61 and 21.54 ppm [32,60]. Figure 2b shows the expanded plot of the methylene region for the four poly(propylene-*co*-α-olefin) copolymers. The peaks at 46.27 and 46.56 ppm was assigned to the PPPP and PPPY sequences, respectively (Figure 2b). It is noted that no resonance peaks of the PYYP tetrad in the spectrum was found—indicating the absence of continuous incorporation and isolated distribution of α-olefin within the polymer chain even in the copolymers with a higher α-olefin comonomer content.

The melting temperature ™ of the PP main chain in these poly(propylene-*co*-α-olefin)s were determined by DSC and listed in Table 1 and the typical melting curves were shown in Figure S2 (Supplementary Materials). The *T*_m_ values decreased with the increase of the comonomer incorporation. This tendency was observed for all of the copolymer samples. The length of the regular sequence of polypropylene is reduced by the comonomers which are rejected from the crystalline part, thus, leading to a reduction of the size and concentration of crystals. Thus, the imperfect crystals in these PP-based elastomers result in lower *T*_m_ compared to that of the PP homopolymer. This observation also demonstrates that all the α-olefin comonomers can induce a comparable effect in shortening the regular isotactic sequences, leading to a decrease of melting temperature. Interestingly, for the P/C20 with about 18 mol.% eicosene units, two melting temperatures corresponding to the *i*PP backbone and side chain was observed at ca *T*_m1_ = 80 °C and *T*_m2_ = 16 °C, respectively. Meanwhile, two crystallization peaks were observed at 15 and 2 °C, which may be attributed to the crystallization peaks of the *i*PP main chain and side chain, respectively. These results further prove that the presence of the α-olefin comonomers sequences have a significant influence on the thermal behaviors of the PP-based elastomer. This will definitely affect the physical property of the materials.

### 4.3. Mechanical Properties

**Figure 3:**
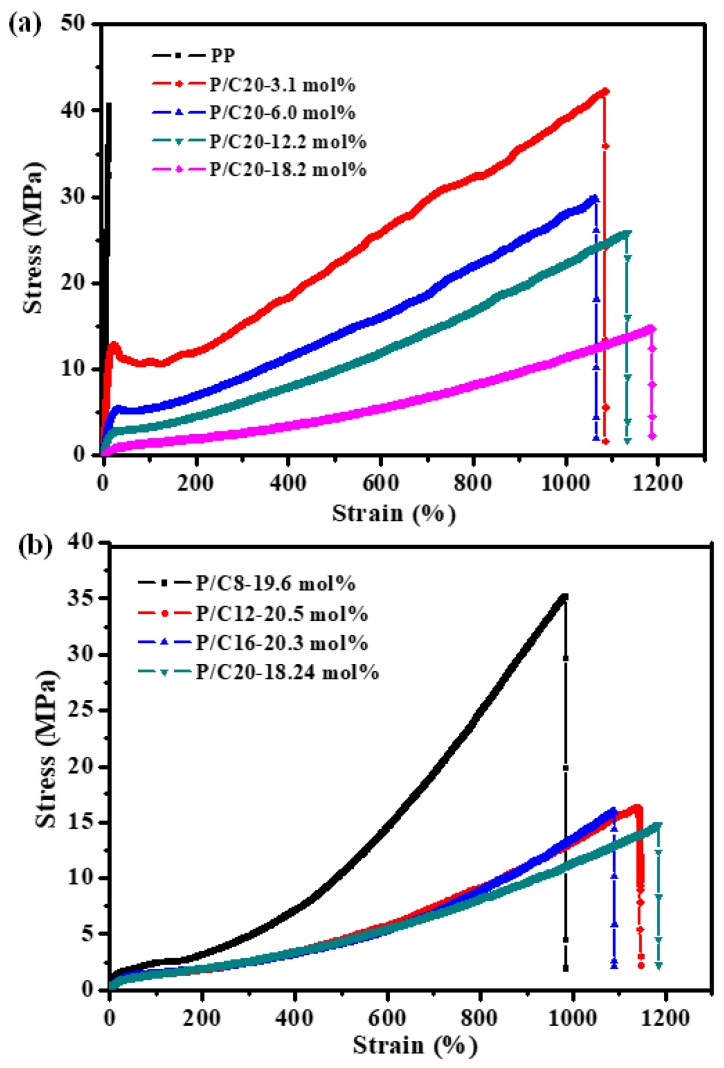
Stress-strain curves of the typical copolymers: (**a**) P/C8 and other poly (propylene-*co*-α-olefin) s with similar comonomer incorporation, (**b**) P/C20 with different 1-eicosene incorporation.

It was proved that the incorporation of higher α-olefin (side chain) could reduce the crystallization tendency of *i*PP. The mechanical properties of *i*PP are largely determined by its crystal structure, which in turn depends on the chain microstructure. Thus, we performed strain-stress tests for the copolymers to study the influence of the random copolymers with different comonomer chain lengths and incorporation (side chain length and density) on the mechanical property. Figure 3 shows the representative stress-strain curves of the compression molded films from the poly (propylene-*co*-α-olefin)s. The typical values of tensile properties, including Young’s modulus (*E*), stress (*σ*_b_) and strain (*ε*_b_) at break and stress (*σ*_y_) and strain (*ε*_y_) at yield point are summarized in Table 2. We concentrate on figuring out the relationship between chain microstructure and mechanical performances. The stress-strain curves of the P/C20 (Figure 3a) indicated that the comonomer incorporation exerted great influence on the mechanical properties of the copolymers. All of the copolymer samples, even the samples containing only 3 mol.% comonomer units, showed a significant enhancement of the ductility and flexibility compared to highly stereo-regular isotactic PP produced by the dimethyl (pyridylamido) hafnium catalyst. At the same time, both Young’s modulus and the stress values at yield point gradually decrease with an increase of the comonomer incorporation. The copolymer samples with low comonomer incorporation (less than 6 mol.%) behave as thermoplastic, as evidenced by the presence of yield point and formation of the sharp neck. The high value of tensile strength and the low values of stress at yielding are related to the strong strain hardening experienced by the samples at high deformation. In contrast, although a yield point was observed, the yield stress is much small in less crystalline samples containing about 12 mol.% comonomer units (Figure S3 (Supplementary Materials) in supporting information). The copolymer sample with a 1-eicosene incorporation of about 18 mol.% shows a typical elastomeric behavior. They deformed more homogeneously with ill-defined yield point, but the strain-hardening is obvious. Similar evolution trends in mechanical properties were also observed in the copolymers P/C8, P/C12 and P/C16 with the increase of monomer incorporation.

In the case of poly (propylene-co-α-olefin) s with different side chain lengths, P/C8-19.6 mol.% showed significantly higher Young’s moduli and tensile strength than those of P/C12-20.3 mol.%, P/C16-20.5 mol.% and P/C20-18.24 mol.% (Figure 3b). However, when the side chain length was increased from 10 to 18 (C12, C16 and C20), the influence of the side chain length on the stress-strain curve becomes negligible. Zhang et al. previously reported that the side chain should be regarded as defects, and the critical length for the side chain to form cocrystallization with PE backbone is 10 carbons [61]. In our case, the lattice of PE side chain and *i*PP backbone is quite different, which imply that all of the PE side chains in poly(propylene-co-α-olefin)s exist as defects for crystals of the *i*PP backbone. As a result, the perfect lamellar structure was formed in P/C8 leading to improved mechanical property. As for the copolymers containing side chain of 10, 14, 18 carbons (C12, C16, C20), the crystallization of side chain had very similar effects on the formation of *i*PP crystals. Therefore, the copolymers with a side chain length longer than 10 carbons (P/C12, P/C16, P/C20) exhibited a similar stress-strain behavior during the deformation process.

**Figure 4:**
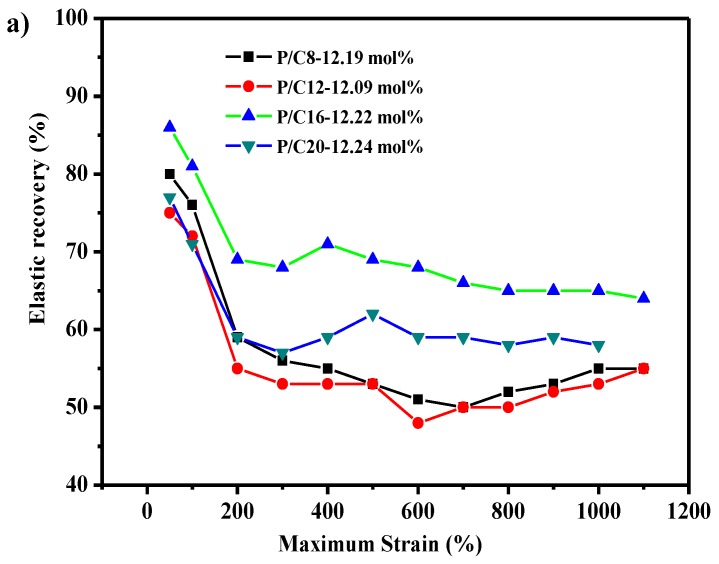

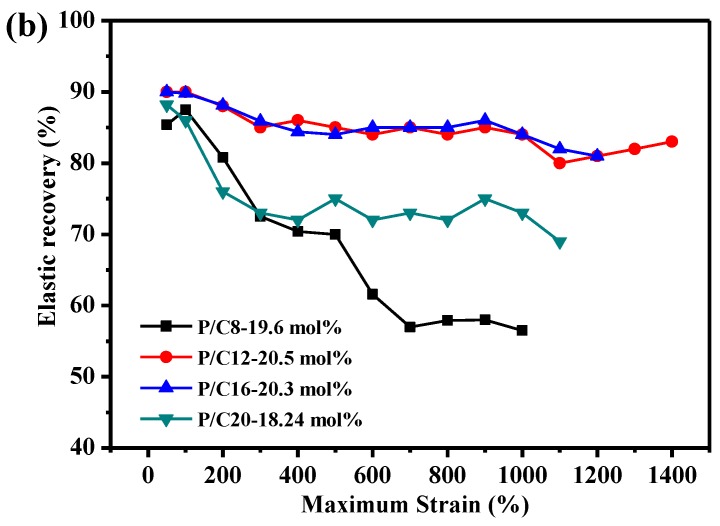
Elastic recovery of the poly (propene-*co*-α-olefin) copolymers containing about 12 mol.% (**a**) and 20 mol.% (**b**) of high α-olefin comonomers.

It is worth emphasizing that the poly (propene-*co*-α-olefin) s (containing 12.0 and 20.0 mol.% α-olefin units) with poorly crystalline presented typical stress-strain curves of elastomeric materials, exhibiting a small yielding and high deformation at the break. Besides, strain-hardening at high deformation was also observed. The DSC analysis has clearly shown that these samples possess detectable *T*_m_, indicating the existence of small crystalline domains. The small crystalline domains will act as physical cross-links in the amorphous matrix and endow the materials with attractive elastomeric properties. The elasticity is the ability of a material to return to its initial state once the force removed. The step cycle tensile tests are the most effective method to characterize the elasticity. Consequently, we measured the elastic recovery of poly (propene-co-α-olefin) copolymers. Firstly, we measured the elastic recovery of poly (propene-*co*-α-olefin) copolymers with about 12 mol.% α-olefin units, and the tensile curves were shown in Figure S4 (Supplementary Materials). Figure 4a summarized the values of elastic recovery for poly (propene-*co*-α-olefin) copolymers with about 12.0 mol.% α-olefin units. As shown in Figure 4a, the elastic recovery quickly decreased when the deformation was increased from 50 to 200%. The copolymer P/C16 with about 12 mol.% comonomer units exhibited the best elastic recovery among the copolymers of the similar comonomer incorporation during the whole deformation process. Secondly, we further measured the elastic recovery of poly (propene-*co*-α-olefin) copolymers with about 20 mol.% α-olefin units, and the tensile curves were shown in Figure S5 (Supplementary Materials). Figure 4b presented the values of elastic recovery for poly (propene-*co*-α-olefin) copolymers with about 20.0 mol.% α-olefin units. Interestingly, when the comonomer incorporation increased, elastic recovery performance of the P/C16 copolymers enjoyed an impressive improvement compared to that of P/C16 copolymers with 12 mol.% α-olefin units, exhibiting higher than 80% over the whole range of deformation. The elastic recovery of the copolymer with the same level of monomer incorporation, but different side chain length was further investigated to reveal the effects of chain length on the mechanical properties and obtain PP-based elastomer with high performance. As shown in Figure 4b, the elastic recovery of PP-based elastomer was greatly improved when the length of the side chain increased from C8 to C16. However, the further increase of side chain length from C16 to C20 leads to a significant decrease in elastic recovery during deformation. These results imply that the side chain with a certain length promises a good recover property.

**Figure 5:**
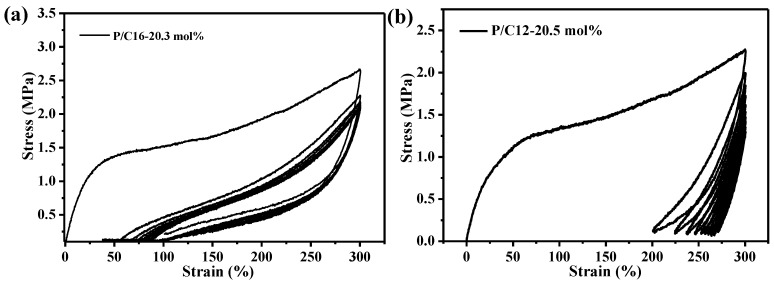
Hysteresis tests for P/C16-20.3 mol.% (**a**) and P/C12-20.5 mol.% (**b**).

Moreover, to deeply understand the elastic performance of the PP-based elastomer, we also conducted the hysteresis tests for the typical copolymers P/C12 and P/C16 with the similar incorporation of 20 mol.% commoners. The samples were cyclically loaded and unloaded ten times to 300% strain, as shown in Figure 5. Although the P/C16 copolymer exhibited a certain amount of unrecovered strain after the first cycle because of a permanent structural change to some extent, a pretty good elastomeric performance was still achieved with only a minimum deformation on each subsequent cycle. Even after 10 cycles of tensile recovery, the unrecovered strain from 300% is less than 40%, which is totally different from that of the copolymer P/C12 copolymer. As for the P/C12 copolymer, a large amount of unrecovered strain after the first cycle and significant deformation on each subsequent cycle were observed, indicating a relative poor elastic recovery. In summary, copolymer P/C16 with 20.3 mol.% monomer incorporation and ideal side chain length is an outstanding elastomeric material with desirable mechanical performance.

**Scheme 2:**
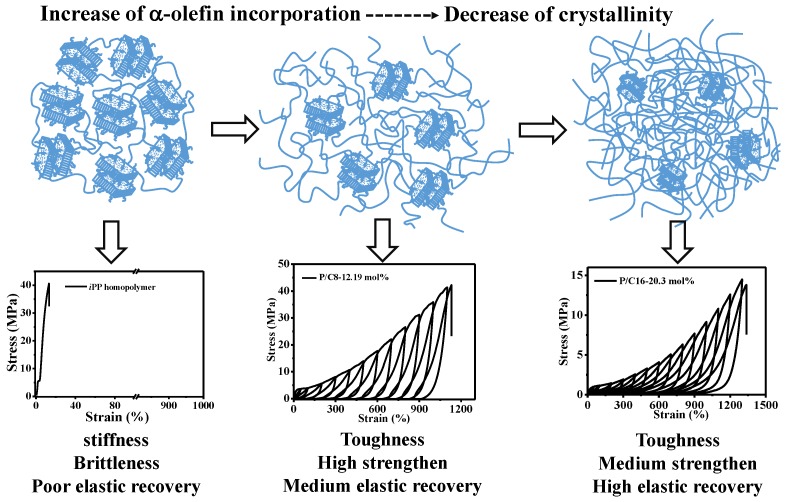
The preparation PP-based elastomer through copolymerization of propene and α-olefins.

We envisioned that the synergistic effect of the physical cross-links acted by the small crystalline domains and the appropriate chain entanglement density is the key mechanism for the good physical performance of the PP-based elastomers. As illustrated in Scheme 2, the incorporation of α-olefin units into the PP backbone results in a decrease in crystallinity of copolymer, which enables the copolymers varying from brittle plastic to toughening elastomer. The presence of small crystalline anchors was believed to prevent the viscous flow of the chains and give a typical elastomeric behavior with a remarkable value of the strength. In addition, the chain entanglement density in the amorphous phase, which is mainly affected by the molecular weight and architecture, will also contribute the elastic recovery after deformation [62,63,64,65,66]. The poly (propene-*co*-α-olefin) copolymers with 12 mol.% incorporation showed a good tensile property. However, the crystalline side chain domain might experience plastic deformation at high tensile stress during elongation, resulting in a lower elastic recovery at high deformation. In contrast, as for the PP-based elastomers containing high comonomer incorporation (20 mol.%) and longer side chain length, the crystallinity and entanglement density in amorphous domains achieve a perfect balance, thus, leading to a significant improvement of the value of elastic recovery in the whole stretching process.

## 5. Conclusions

Activated by [Ph_3_C] [B (C_6_F5)_4_], dimethylpyridylamidohafnium complex exhibited excellent performance (catalytic activity is up to 6 × 10^7^ g/mol_Hf_⋅h⋅atm) in the copolymerization of propene with higher α-olefin, including C8, C12, C16 and C20. PP copolymers with high molecular weight (up to 160 × 10^4^ g/mol) and unimodal distributions (PDI < 1.6) were easily obtained under mild condition. The ^13^C NMR analysis showed the isolate distribution of α-olefin and the absence of continuously incorporated α-olefin sequence. Thus, the randomly distributed α-olefin units shortened the average length of *i*PP sequence and resulted in a decrease in *T*_c_ values of the copolymers. Two crystallize peak assigned to the PP backbone and side chain, respectively, was observed in the P/C20 copolymers. All of the copolymers exhibited higher ductility and flexibility than *i*PP because of the reduced crystallinity. The copolymers with about 20 mol.% monomer incorporation contains exhibited elastomeric behavior, in which a small amount of crystalline (serve as physical cross-linking) was dispersed in the large fraction of amorphous matrix. The side chain length has important effects on the elastic recovery. The elastic recovery of P/C8 and P/C20 with about 20% monomer incorporation decreased significantly at higher strain, while the copolymer P/C16 and P/C12 exhibited high elastic recovery (higher than 80%) in the whole range of deformations. Subsequent hysteresis tests revealed a large amount of unrecovered strain after the first cycle and significant deformation on each subsequent cycle in the copolymer P/C12. Although nearly 35% unrecovered strain occurred after the first cycle, minimum deformation and good recovery were observed on each subsequent cycle. Thus, P/C16 copolymers with about 20 mol.% hexadecane units were proved to be an outstanding PP-based elastomeric material.

## Figures and Tables

**Table 1:** The data of copolymerization of propylene with higher α-olefins *^a.^*

Entry	Comonomer (mmol)	Incorp *^b^* (mol.%)	Act *^c^*	*M*_w_*^d^* (kg/mol)	PDI *^d^*	*T*_m_*^e^* (°C)	*X*_c_*^f^* (%)
1			3.0	295	2.0	160	54
2	1-octene(1.50)	3.1	3.6	335	1.6	117	26
3	1-octene(3.00)	6.0	4.1	417	1.6	92	19
4	1-octene(4.50)	12.2	4.7	467	1.4	79	14
5	1-octene(6.00)	19.6	5.0	519	1.4	46	9
6	1-dodecene(1.50)	3.0	4.0	586	1.4	118	28
7	1-dodecene(3.00)	5.9	4.5	678	1.5	101	18
8	1-dodecene(4.50)	12.1	4.7	651	1.4	46	11
9	1-dodecene(6.00)	20.5	5.1	960	1.4	33	9
10	1-hexadecene(1.50)	3.1	4.2	603	1.5	115	24
11	1-hexadecene(3.00)	6.2	4.7	696	1.4	95	18
12	1-hexadecene(4.50)	12.2	4.9	1251	1.6	46	11
13	1-hexadecene(6.00)	20.3	5.2	1416	1.5	34	10
14	1-eicosene(1.50)	3.1	4.6	689	1.4	120	23
15	1-eicosene(3.00)	6.0	5.1	1482	1.5	115	16
16	1-eicosene(4.50)	12.2	5.3	1505	1.4	114	10
17	1-eicosene(6.00)	18.2	5.7	1678	1.4	80	8

***^a^*** Cat. Hf 1 μmol, [Ph_3_C] [B(C_6_F_5_)_4_] 2 μmol, Al*^i^*Bu_3_ 0.20 mmol, propylene 1 atm, toluene solution, *V*_total_ = 40 mL, at 25 °C. *^**b**^* Comonomer incorporation (mol.%) was established by ^13^C NMR Spectroscopy, monomer incorporation = 1n(A43.7+A35.9+A33.5+A31.9+A30.3+A29.7+A29.3+A26.8+A22.6+A13.9)1n(A43.7+A35.9+A33.5+A31.9+A30.3+A29.7+A29.3+A26.8+A22.6+A13.9)+13(A46.2+A28.6+A21.6) ×100%, n is the carbon number of higher α-olefins. ***^c^*** Catalytic activity: 10^7^ g_polymer_⋅mol_Hf_^−1^⋅h^−1^. ***^d^*** Weight-average molecular weights and polydispersity indices determined by SEC with light scattering detector at 150 °C in 1,2,4-C_6_Cl_3_H_3_. ***^e^*** Melting temperature and heat of PP main chain determined by DSC at a rate of 10 °C/min, the *T*_m_ was obtained from the second heating cycle. ***^f^*** *χ*_c_ (crystalline degree of PP main chain) = Δ*H*_m_/Δ*H*_m_^0^ × ω(PP). Δ*H*_m_^0^ = 207 J/g for 100% crystalline isotactic polypropylene.

**Table 2:** The value of mechanical properties for random poly (propylene-co-α-olefin)s.

Sample	*E* (MPa)	*σ*_y_ (Mpa)	*ε*_y_ (%)	*σ*_b_ (Mpa)	ε_b_ (%)
*i*PP	600 ± 15			40.5 ± 1	13 ± 0.5
P/C8-12.19	25.3 ± 2.2	3.7 ± 0.1	35 ± 1	38 ± 3	950 ± 20
P/C8-19.60	13 ± 0.1			35 ± 2	1000 ± 40
P/C12-12.09	4.0 ± 0.2	3.2 ± 0.1	34 ± 1	33 ± 3	1100 ± 40
P/C12-20.50	2.2 ± 0.2			17 ± 1	1150 ± 20
P/C16-12.22	3.8 ± 0.1	1.6 ± 0.1	32 ± 1	28 ± 3	1100 ± 20
P/C16-20.30	3.2 ± 0.4			18 ± 2	1100 ± 30
P/C20-3.06	157 ± 5	12.9 ± 0.3	21 ± 2	30 ± 2	1100 ± 20
P/C20-5.96	45 ± 2	5.3 ± 0.1	24 ± 1	26 ± 1	1050 ± 30
P/C20-12.24	2.7 ± 0.1	2.9 ± 0.1	32 ± 1	25 ± 3	1140 ± 50
P/C20-18.24	1.8 ± 0.1			15 ± 1	1200 ± 30

## Supplementary Materials

Data, including characterization of the polymer samples, is available online. https://www.mdpi.com/2073-4360/12/1/89/s1, Figure S1: ESI-MS spectra of propylene/1-octene copolymerization filtrate (tested under humid aerobic conditions). Figure S2: The DSC curves of the typical copolymers: poly(propylene-*co*-1-hexadecene)s and other poly(propylene-*co*-α-olefin)s with various α-olefin incorporation. Figure S3: Stress-strain curves of the typical copolymers: poly(propylene-*co*-1-octene)s and other poly(propylene-*co*-α-olefin)s with similar comonomer incorporation (~12 mol.%). Figure S4: Cyclic tensile test curves of poly(propylene-*co*-α-olefin) with similar comonomer incorporation (~12 mol.%) under a maximum strain from 50% to 1200%. Figure S4: Cyclic tensile test curves of poly(propylene-*co*-α-olefin) with similar comonomer incorporation (~20 mol.%) under a maximum strain from 50% to 1200%.
