# Supplementary material for: Copolymerization of Propylene with Higher α-Olefins by a Pyridylamidohafnium Catalyst: An Effective Approach to Polypropylene-Based Elastomer
Source: Polymers (Basel). 2020 Jan 3;12(1):89. doi: 10.3390/polym12010089 (PMC7023639; doi:10.3390/polym12010089)
Supplement: Supplementary file 1 [file polymers-12-00089-s001.pdf]

Supporting Information

Article

Copolymerization of Propylene with Higher α-Olefins by a Pyridylamidohafnium Catalyst: An Effective Approach to Polypropylene-Based Elastomer

Fei Yang [1], Xiaoyan Wang [2,*], Zhe Ma [1], Bin Wang [1,*], Li Pan [1] and Yuesheng Li [1]

[1] Tianjin Key Laboratory of Composite & Functional Materials, School of Materials Science and Engineering, Tianjin University, Tianjin 300350, China; fyang@tju.edu.cn (F.Y.); zhe.ma@tju.edu.cn (Z.M.); lilypan@tju.edu.cn (L.P.); ysli@tju.edu.cn (Y.L.)

[2] State Key Laboratory of Organometallic Chemistry, Shanghai Institute of Organic Chemistry, Chinese Academy of Sciences, 345 Lingling Lu, Shanghai 200032, China

* Correspondence: wangxiaoyan@sioc.ac.cn (X.W.); binwang@tju.edu.cn (B.W.)

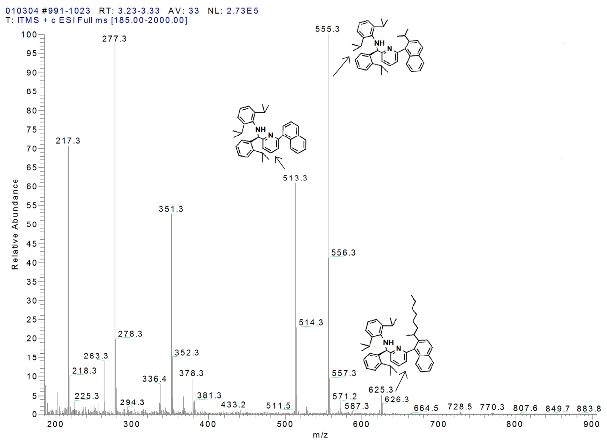

Figure S1. ESI-MS spectra of propylene/1-octene copolymerization filtrate (tested under humid aerobic conditions).

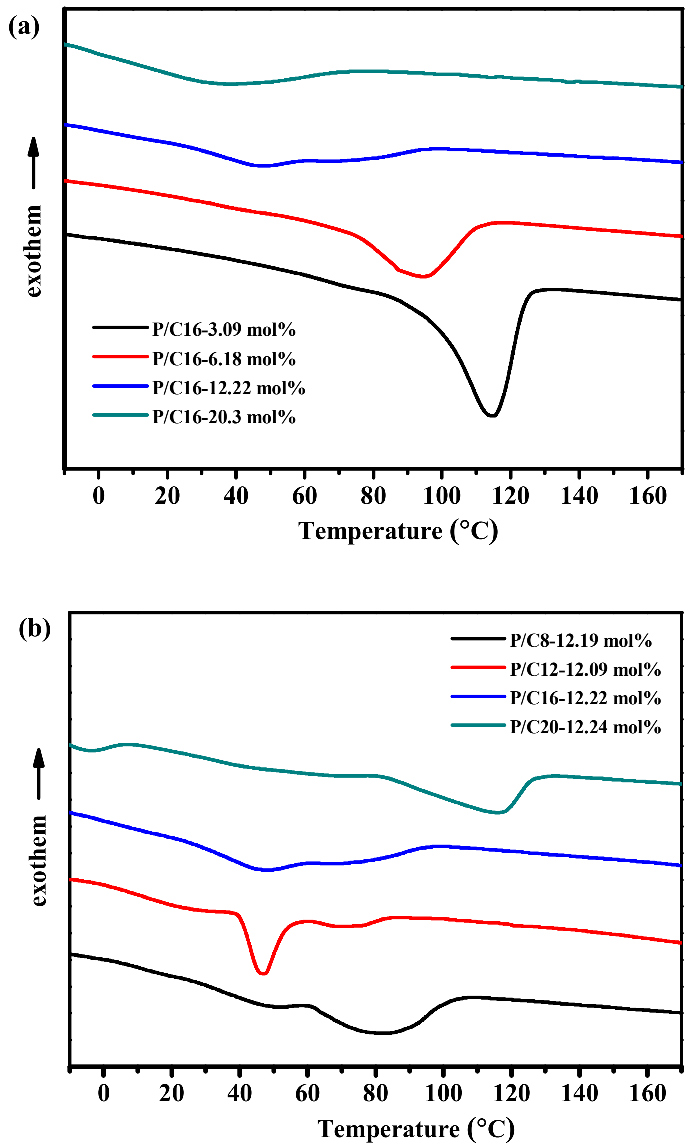

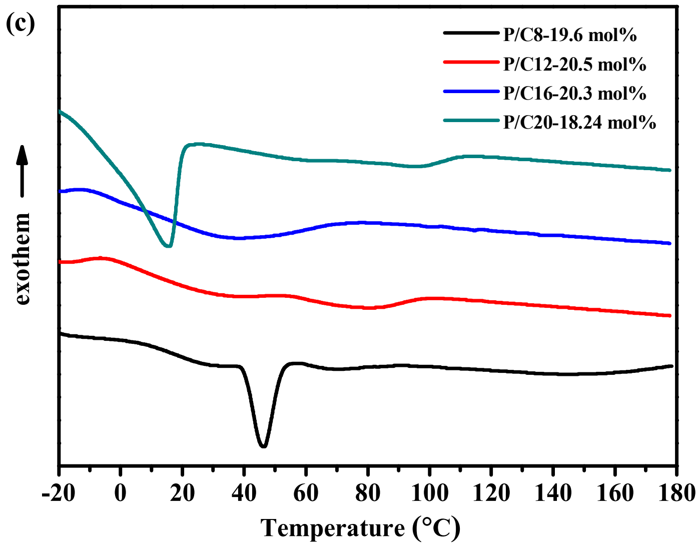

Figure S2. The DSC curves of the typical copolymers: poly(propylene-*co*-1-hexadecene)s and other poly(propylene-*co*-α-olefin)s with various α-olefin incorporation.

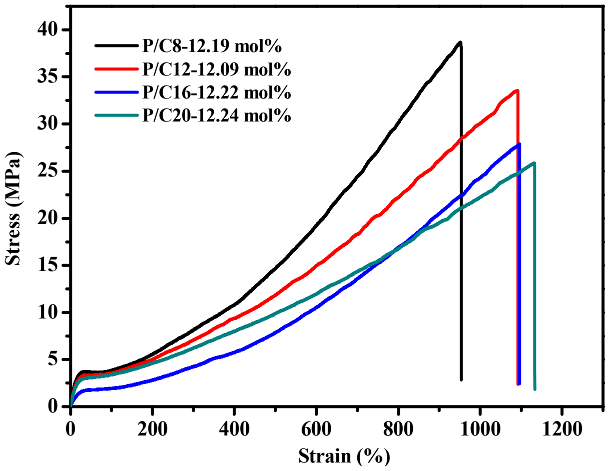

Figure S3. Stress-strain curves of the typical copolymers: poly(propylene-*co*-1-octene)s and other poly(propylene-*co*-α-olefin)s with similar comonomer incorporation (~12 mol%).

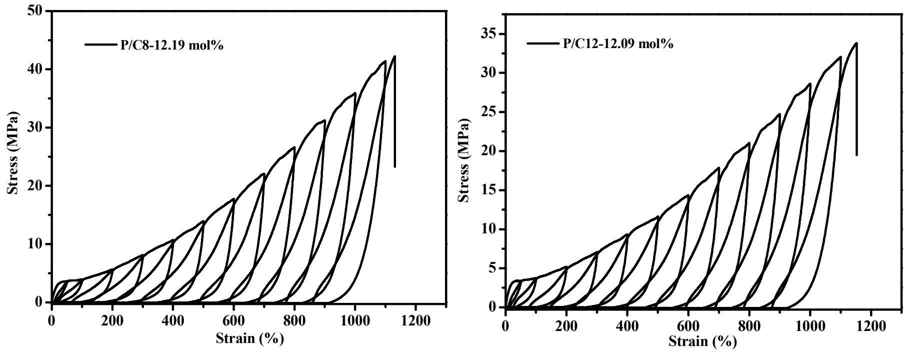

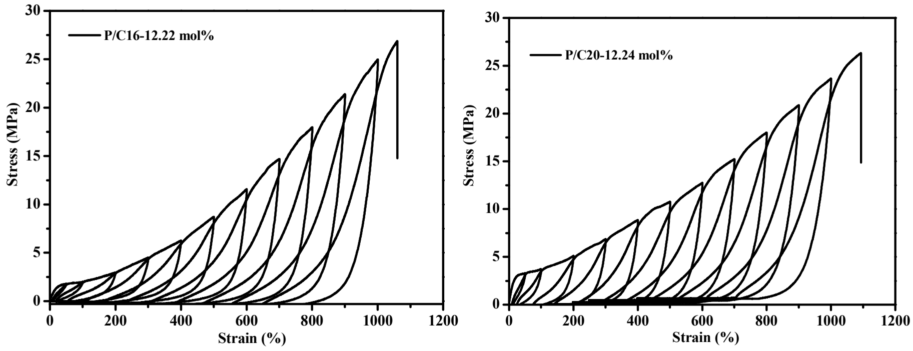

Figure S4. Cyclic tensile test curves of poly(propylene-*co*-α-olefin) with similar comonomer incorporation (~ 12 mol%) under a maximum strain from 50% to 1200%.

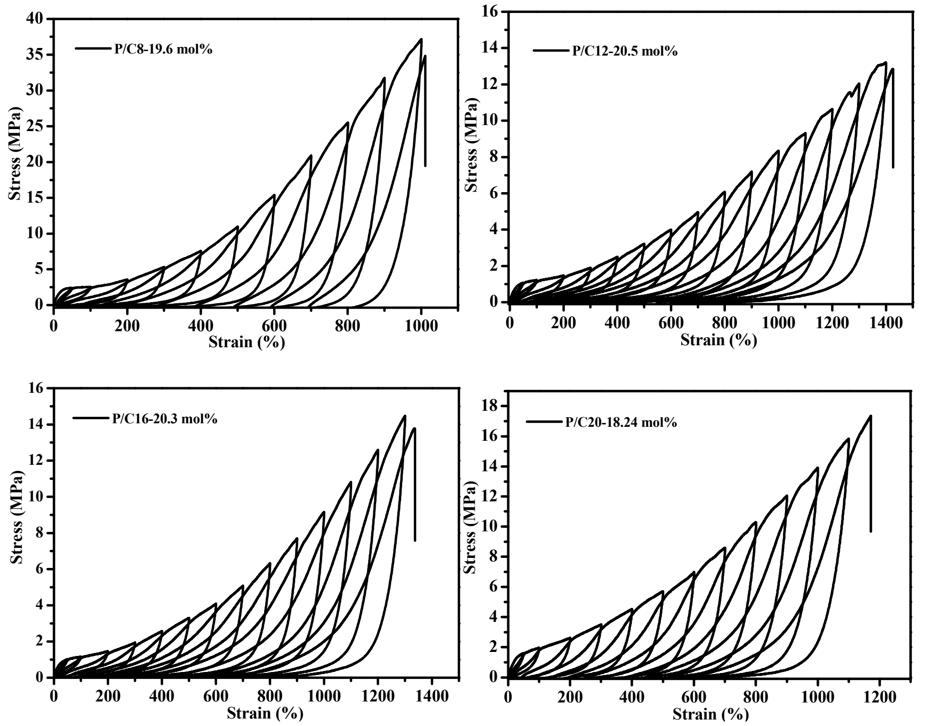

Figure S5. Cyclic tensile test curves of poly(propylene-*co*-α-olefin) with similar comonomer incorporation (~ 20 mol%) under a maximum strain from 50% to 1200%.
